# Supplementary material for: Oxidative insult can induce malaria-protective trait of sickle and fetal erythrocytes
Source: Nat Commun. 2016 Nov 8;7:13401. doi: 10.1038/ncomms13401 (PMC5105170; doi:10.1038/ncomms13401)
Supplement: Supplementary Information — Supplementary Figures 1-2, Supplementary Tables 1-2 and Supplementary References [file ncomms13401-s1.pdf]

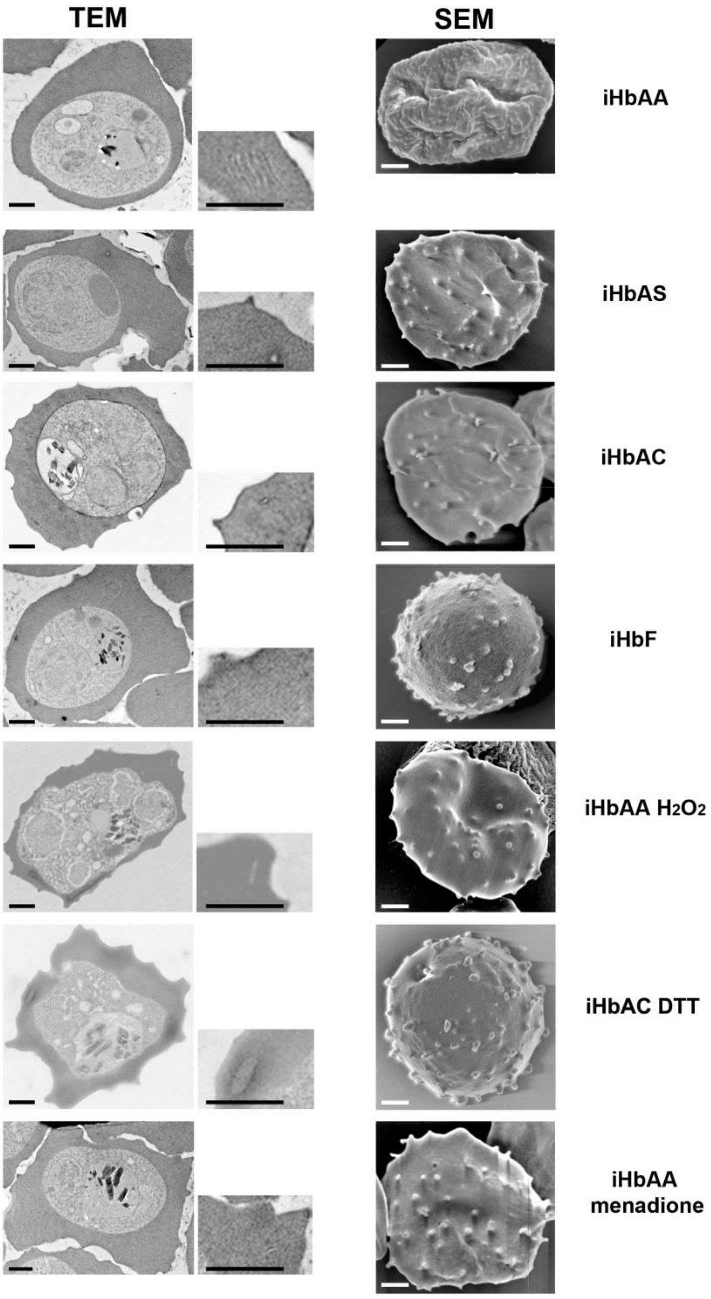

Supplementary Figure 1. Differential morphological features of parasitized erythrocytes containing different haemoglobin variants and following different treatments prior to infection with the *P. falciparum* strain FCR3^{CSA}. Transmission (TEM) and scanning (SEM) electron microscopic images are shown. TEM images show morphology of knobs and Maurer's clefts. The inserts depict representative Maurer's clefts and knobs. SEM images show density, sizes and shapes of knobs on the surface of infected erythrocytes. Scale bars, 2 µm.

a

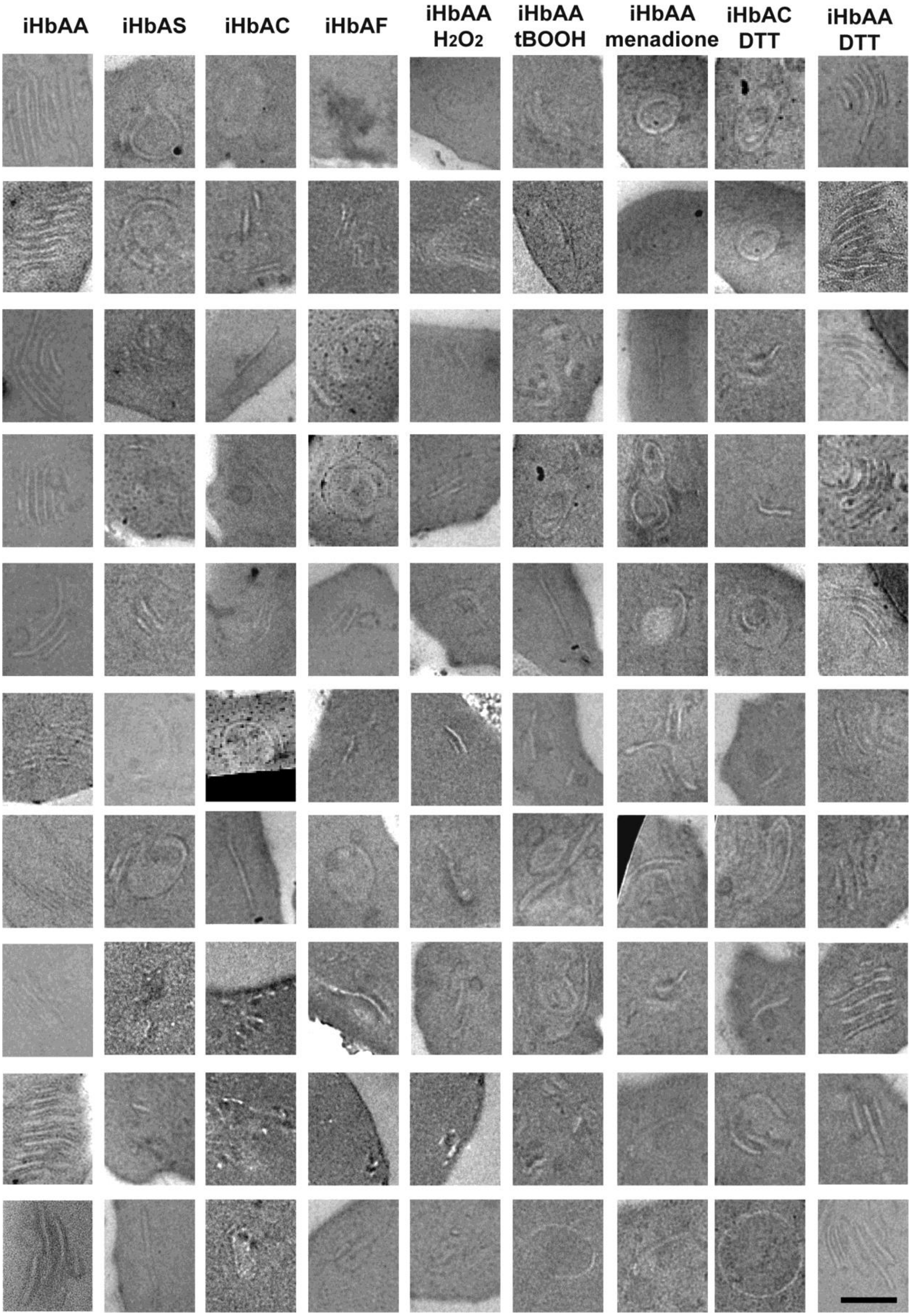

iHbAA
iHbAS
iHbAC
iHbAF
iHbAA H2O2
iHbAA tBOOH
iHbAA menadione
iHbAC DTT
iHbAA DTT

b

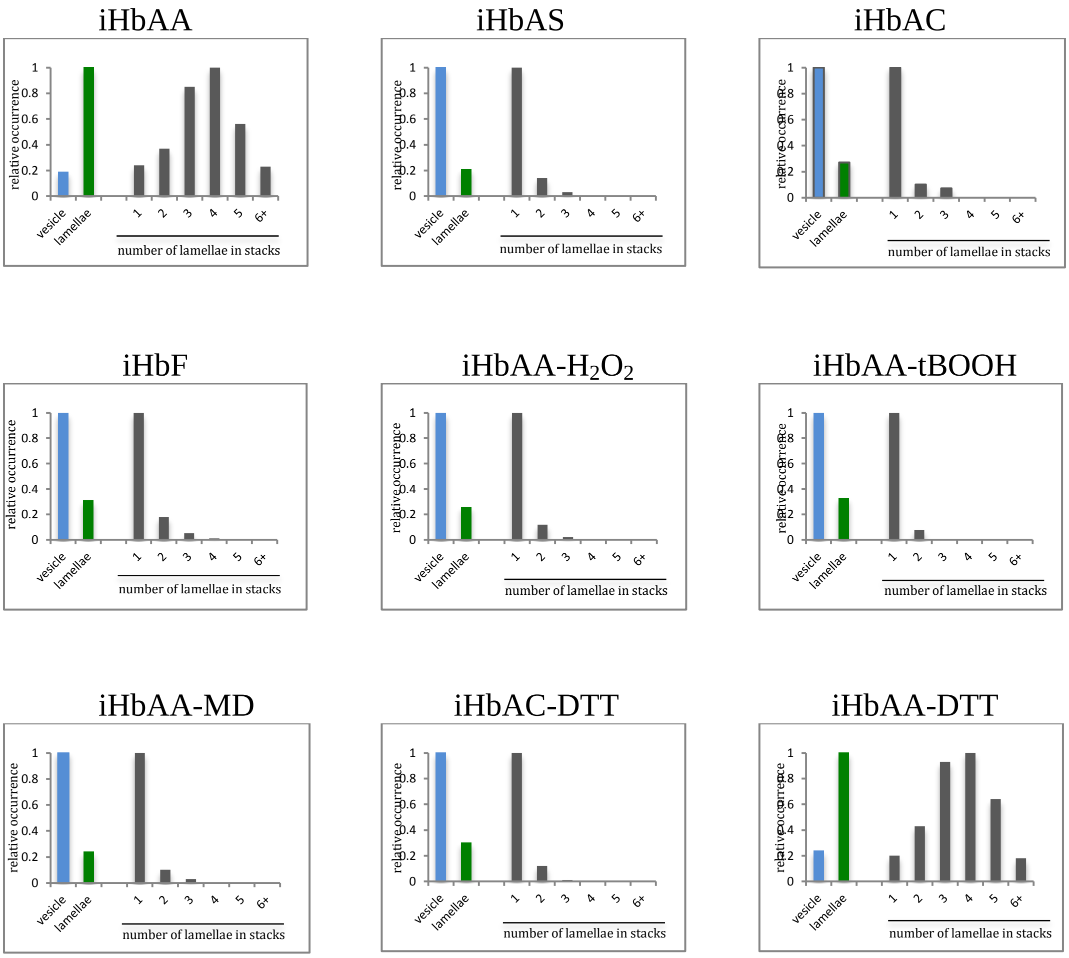

Supplementary Figure 2. Maurer's clefts morphology of parasitized erythrocytes containing different haemoglobin variants and following different treatments prior to infection with the *P. falciparum* strain FCR3^{CSA}. (**a**) A gallery of 2D TEM images of Maurer's clefts as typically observed in parasitized HbAA, HbAS, HbAC, HbF erythrocytes, and in erythrocytes pretreated with H_2O_2, tert-butyl hydroperoxide (tBOOH), DTT, and menadione. Scale bar: 1 µm. (**b**) Shape distributions of Maurer's clefts in wild type and "protective" conditions, showing the ratio of vesicles (blue bars) versus lamellae (green bars). The lamellar shape dominates in parasitized HbAA erythrocytes (HbAA and HbAA-DTT), whereas the vesicle-like shape dominates in all "protective" conditions. Grey bars indicate a distribution of the number of lamellae in Maurer's clefts stacks (ratios). For each condition at least 100 Maurer's clefts were analysed.

Supplementary Table 1: Semi-quantitative measurements of the Maurer's clefts membranous compartments in various parasitized erythrocytes. Average volumes and membrane surface areas were measured in 100 nm thick TEM sections, and extrapolated to a volume of a whole cell, using previously described protocols [1, 2]. Note the volume to surface area ratio of Maurer's clefts doubled in all "protective" conditions as compared to parasitized HbAA erythrocytes (WT).

Condition	# Lamellae/ EM section	Lamellae volume/ EM section[a] (μm^3)	Lamellae surface/ EM section[a] (μm^2)	# Lamellae/ cell[b]	# Lamellae ratio to WT	Lamellae volume/ cell[b] (μm^3)	Lamellae surface/ cell[b] (μm^2)	Volume/ surface ratio	Volume gain as ratio to WT
iHbAA	0.34	0.0026	0.097	54[c]	1.0	0.48	18.0	0.027	1.0
iHbAS	0.54	0.0053	0.091	85	1.6	0.98	16.9	0.058	2.2
iHbAC	0.57	0.0049	0.097	90	1.7	0.91	18.1	0.050	1.9
iHbF	0.56	0.0047	0.094	88	1.6	0.88	17.6	0.050	1.9
iHbAA-H_2O_2	0.62	0.0050	0.101	97	1.8	0.93	18.8	0.049	1.9
iHbAA-tBOOH	0.63	0.0045	0.094	98	1.8	0.84	17.5	0.048	1.8
iHbAA-menadione	0.64	0.0052	0.091	101	1.9	0.97	17.0	0.057	2.1
iHbAC-DTT	0.56	0.0050	0.094	87	1.6	0.92	17.5	0.053	2.0
iHbAA-DTT	0.31	0.0023	0.094	49	0.9	0.44	17.4	0.025	0.9

[a] an average volume and surface area of membranous compartments of Maurer's clefts (lamellae and vesicles) in 100 nm thick EM sections;
[b] interpolated to an average volume of an infected erythrocyte cytoplasm; cell dimension in Z: 12.5 µm; i.e. 125 sections / cell; Z dimension of lamellae: 345 nm (as an average in X and Y), i.e. 1 lamella was observed in 2.9 cell sections of HbAA
[c] corresponds to 12.3 Maurer's clefts / cell for HbAA (on average 4.3 lamellae / Maurer's cleft)

Supplementary Table 2. Primers used for quantification of *var2csa* and *cyclophilin* mRNA.

Target	Primer Sequence (5' -> 3')
PFE0505w_F (cyclophilin_F)	AAACGGGAGATCCTTCAGGT
PFE0505w_R (cyclophilin_R)	AAGGACATGGGACAGTGGTT
PFL0030c_ex_F (var2csa_F)	GACGCGAAACGAAACCGTAA
PFL0030c_ex_R (var2csa_R)	ACTACTTGGGCCACAATTTTTTG

Supplementary References

1. Griffiths, G., Lucocq, J. M. & Mayhew, T. M. Electron microscopy applications for quantitative cellular microbiology. *Cell Microbiol* **3**, 659-668 (2001).

2. Prakash, Y. S., Smithson, K. G. & Sieck, G. C. Application of the Cavalieri principle in volume estimation using laser confocal microscopy. *Neuroimage* **1**, 325-333 (1994).
